# Long-term use of calcium channel blocking drugs and breast cancer risk in a prospective cohort of US and Puerto Rican women

**DOI:** 10.1186/s13058-016-0720-6

**Published:** 2016-07-05

**Authors:** Lauren E. Wilson, Aimee A. D’Aloisio, Dale P. Sandler, Jack A. Taylor

**Affiliations:** Epidemiology Branch, National Institute of Environmental Health Sciences, National Institutes of Health, Research Triangle Park, NC 27709 USA; Social & Scientific Systems, Inc., Durham, NC 27703 USA

**Keywords:** Calcium channel blockers, Antihypertensive drugs, Breast cancer risk

## Abstract

**Background:**

In a recent case–control study, long-term use of calcium channel blocking drugs was associated with a greater-than-twofold increased breast cancer risk. If prospectively collected data confirm that calcium channel blocker use increases breast cancer risk, this would have major implications for hypertension treatment. The objective of this study was to determine whether women using calcium channel blockers for 10 years or more were at increased risk of developing breast cancer compared with women not using calcium channel blockers.

**Methods:**

The Sister Study is a prospective volunteer cohort study of women from the USA and Puerto Rico designed to evaluate environmental and genetic risk factors for breast cancer. Beginning in 2003, women between the ages of 35 and 74 were recruited. They were eligible to participate if they had a sister with breast cancer but had not been diagnosed with breast cancer themselves. In total, 50,884 women enrolled in the cohort between 2003 and 2009; 50,757 women with relevant baseline data and available follow-up data are included in this study. The exposure of interest is current use of calcium channel blocking drugs and the reported duration of use at entry into the cohort. Secondary exposures of interest were the duration and frequency of use for all other subclasses of antihypertensive drugs. Our main outcome is a self-reported diagnosis of breast cancer during the study follow-up period. With patient permission, self-reported diagnoses were confirmed using medical records.

**Results:**

Results showed 15,817 participants were currently using an antihypertensive drug, and 3316 women were currently using a calcium channel blocker at study baseline; 1965 women reported a breast cancer diagnosis during study follow-up. Using Cox proportional hazards modeling, we found no increased risk of breast cancer among women who had been using calcium channel blockers for 10 years or more compared with never users of calcium channel blockers (HR 0.88, 95 % CI 0.58–1.33).

**Conclusions:**

We saw no evidence of increased risk of breast cancer from 10 years or more of current calcium channel blocker use. Our results do not support avoiding calcium channel blocking drugs in order to reduce breast cancer risk.

**Electronic supplementary material:**

The online version of this article (doi:10.1186/s13058-016-0720-6) contains supplementary material, which is available to authorized users.

## Background

Antihypertensive drugs constitute the most commonly prescribed drug class in the USA. In 2010, Americans filled 678.2 million antihypertensive drug prescriptions [1, 2], and after starting, many individuals continue taking the drug for the rest of their lives [2]. The association between antihypertensives and breast cancer risk has been evaluated in a limited number of studies with mixed results.

A recent case–control study of women aged 55–74 reported a twofold increased risk of breast cancer for women who were current calcium channel blocker users with at least 10 years of use [1]. This increased risk was observed relative to women who had never used antihypertensive drugs, and relative to women using another type of antihypertensive for 10 years or more. They also reported a higher risk increase for breast cancer for women with current use of the nondihydropyridine subclass of calcium channel blockers compared with those using dihydropyridines [1]. Several other case–control studies have also reported increased breast cancer risk in women who used calcium channel blockers [1, 3–5] or with recent use (past 2 years) of immediate-release (short-acting) calcium channel blockers [3] However, case–control studies are limited in their ability to establish causal risk associations; collecting exposure information after disease diagnosis may lead to recall bias. Prospective studies that assess exposure before disease diagnoses occur are effective at minimizing biases related to differential exposure recall or possible medication changes following breast cancer diagnosis and treatment. Prospective studies may also provide a more accurate picture of exposures during the at-risk period. A number of prospective cohort studies have reported no association between breast cancer and antihypertensive use in general or by drug subclass [6–13]. However, uncertainty about calcium channel blocker use and breast cancer risk remains; many of these prospective studies have been limited by small numbers of breast cancer cases, failure to examine drug subclasses, or short durations of antihypertensive use.

Contemporary prospective cohort studies with many long-term calcium channel blocker users are needed to determine whether breast cancer risk is associated with long-term use of these drugs. Although two recent cohort studies with large sample sizes did not detect increased risk of breast cancer among users of calcium channel blockers [12, 13], studies of the general population might not accurately capture elevated risks that may be amplified in women with family histories of breast cancer through interaction with other risk factors [14].

The Sister Study is a prospective cohort study of 50,884 women who had a sister with breast cancer and thus are at increased risk of developing breast cancer themselves. This study was designed specifically to capture possible interactions between environmental and lifestyle risk factors such as antihypertensive drug use, and the genetic risk factors underlying familial breast cancer risk. These data were collected in a time period when long-term antihypertensive use was increasingly common among women aged 50 and older, and therefore provide a unique opportunity to evaluate the effect of long-term calcium channel blocker use on breast cancer risk in a higher-risk population of women.

## Methods

### Study population

The Sister Study is a volunteer cohort of women recruited through media and community outreach across the USA and Puerto Rico. Women were eligible to participate if they had a sister with breast cancer and had never been diagnosed with breast cancer themselves. Between 2003 and 2009, the cohort enrolled 50,884 women and participants completed in-person physical examinations and baseline telephone interviews detailing medical, lifestyle, dietary, and reproductive history. Participants are followed for incident breast cancer diagnoses with annual health updates supplemented with detailed biennial and triennial questionnaires with follow-up response rates ≥94 % [15, 16]. The study was approved by the Institutional Review Board of the National Institute of Environmental Health Sciences, NIH and the Copernicus Group Institutional Review Board. Inclusion in this analysis is covered by the informed consent form all included cohort participants agreed to and signed.

Sister Study participants are surveyed for development of breast cancer annually, and are asked to report a new breast cancer diagnosis between follow-ups. Reported cases are confirmed with medical record retrieval. Self-reports and medical records were also used to compile and confirm information on tumor histology and hormone receptor status. At the time of analysis (Data Release 2.0), approximately 91 % of women with breast cancer consented to retrieval of their medical records. All women who reported a breast cancer diagnosis had an opportunity to disavow their report; women who disavowed their initial report of a breast cancer diagnosis (*N* = 3) are not included as cases in this analysis. Of the 77 % of cases (*N* = 1638) who had records available at the time of analysis, there was close agreement between self-report and the medical record for the diagnosis of breast cancer (99.5 %), classification of an invasive cancer (98.5 %), and ER+ status (99 %), providing a high degree of confidence in the use of self-report for those women with missing medical record data. All available breast cancer diagnoses collected during or prior to the last completed follow-up phase as of 26 April 2013 were included.

Information on use of calcium channel blocking drugs or use of other classes of antihypertensive drugs was obtained from the baseline Sister Study questionnaire. Women were asked a series of questions designed to capture information on all current and past medications for all reported medical conditions. Information on medication use collected in the survey included: the patient’s medical conditions; the name of each drug taken for the reported medical conditions; how frequently each drug was taken per week; and the first and last ages at which the drug was taken. They were also asked to report any additional medications taken that were not associated with the medical conditions they reported. To minimize reporting errors, women were asked to have their current medications on hand during the interview. They were provided booklets with the names of commonly used medications for various conditions such as hypertension prior to the interview to promote accurate reporting of current medications and medications used in the past. Each reported medication was coded by product and class using the Slone Drug Dictionary (Boston University, Boston, MA, USA). Products that contained more than one active ingredient were assigned multiple class codes.

We examined the effect of duration and class of antihypertensive drug use on breast cancer risk. We included use of antihypertensive drugs for all disease indications and reported use without a specific indication listed. Covariates were obtained from the baseline Sister Study questionnaires or measured during examiner at-home visits; these included age, race/ethnicity, underlying medical conditions and medical history, family medical history, history of benign breast conditions, smoking status, alcohol consumption habits, level of reported physical activity, body mass index (BMI) from examiner-measured height and weight, age at menarche, menopausal status, hormonal exposures including hormone replacement therapy and oral contraceptives, selected dietary factors, and history of pregnancy and breastfeeding. Baseline menopause status was determined by a women’s self-reported age at last menstrual period (LMP); a women was considered postmenopausal if 12 months or more had passed since her LMP at baseline, or if her last LMP was determined to have occurred pre baseline based on subsequent reports during study follow-up. Women with hysterectomy or uterine surgeries were considered postmenopausal when they reached the age of 56.

### Statistical methods

We characterized use of calcium channel blockers and other antihypertensive drugs in the cohort using standard contingency table methods with chi-square tests. Univariate and multivariate logistic regression analyses were used to identify potential covariates associated with different levels of current and lifetime antihypertensive use across drug subclasses. All statistical analyses were conducted using SAS 9.3 (Cary, NC, USA).

To determine the association between calcium channel blocker use and breast cancer risk, we calculated hazard ratios (HRs) for strata of lifetime use and incident breast cancer using multivariate Cox proportional hazard regression. Calcium channel blocker use was categorized into five levels: (1) never used calcium channel blocker; (2) former user of calcium channel blocker; (3) current user with less than 5 years of use; (4) current user with 5–10 years of use; and (5) current user with 10 years or more of use. The same stratifications were used for use of any type of antihypertensive drug, as well as for the following classes of antihypertensive drugs: beta-blockers, angiotensin-converting enzyme (ACE) inhibitors, diuretics, angiotensin receptor blockers, and miscellaneous drugs. Calcium channel blockers were also stratified into dihydropyridines (amlodipine, felodipine, nifedipine SR, isradipine, nifedipine, nicardipine) and nondihydropyridines (verapamil, diltiazem, verapamil SR, diltiazem XR). Women who switched from one antihypertensive drug subclass to another subclass before the baseline survey were considered former users for their previous drug subclass, current users for their current subclass, and current users of antihypertensives in general.

Attained age serves as the time scale for the Cox proportional hazards regression models. Participants enter the analysis with their age at study enrollment, and accrue person-time until they either exit at the age of breast cancer diagnosis for cases or the age at their last completed health questionnaire. Participants who failed to respond to their most recent eligible health update were censored at the earliest date among death or the midpoint of the interval between the last completed health update and the end of the window of eligibility for responding to their first missed health update. We ran crude unadjusted Cox proportional hazards regression models as well as a multivariable model with adjustment for the following variables selected as a-priori potential confounders based on existing literature: baseline-measured BMI, race/ethnicity, smoking status, parity, menopause status, age at menarche, history of breast conditions, current drinking level, hormone replacement therapy use, and statin use. We also ran models excluding women who reported prophylactic mastectomy at baseline (*N* = 234), or with adjustment for prophylactic tamoxifen and reloxifene use, but these factors had no material effect on our risk estimates and were excluded from the final model.

As an a-priori hypothesis, we examined risk associated with long-term use of calcium channel blocking drugs, and additionally examined use of antihypertensives in general and use of other subclasses of antihypertensive drugs. We focused on use of calcium channel blockers that lasted for 10 years or more, because this is the length of usage that has recently been associated with breast cancer risk. We also conducted analyses where long-term use was defined less strictly as 5 years or more of use to provide increased statistical power for risk detection. We examined risk associated with long-term calcium channel blocker use by breast tumor characteristics including tumor invasiveness at diagnosis and estrogen receptor status. To make our results as comparable as possible with previously conducted studies, we conducted sensitivity analyses limiting our analytical group to: women who were postmenopausal at study entry; and women who were aged 55 or older at study entry. Additionally, we conducted an active comparator sensitivity analysis in which we limited our analytical group to women who were current users of an antihypertensive drug: for at least 5 years; and for at least 10 years. This analysis allows for direct comparison of long-term calcium channel blocker users with women who were long-term users of another class of antihypertensive drug.

## Results

After excluding women missing relevant baseline or follow-up data or who were diagnosed with breast cancer before completion of enrollment activities, we had an analytical cohort of 50,757 women for this study: 17,068 women (33.6 %) reported ever taking an antihypertensive drug in their lifetime; and 15,817 (31.2 %) were currently using an antihypertensive drug, 3316 of whom were currently using a calcium channel blocker at study baseline. The majority of current antihypertensive users (90.1 %) were postmenopausal at baseline. Demographic characteristics of women who had and had not taken calcium channel blockers are presented in Table 1. On average, women who reported calcium channel blocker use were older than nonusers, with a mean age at study baseline of 60.2 years compared with a mean of 54.5 years among women who had never used calcium channel blockers (*p* < 0.0001). A larger proportion of calcium channel blocker users were black (21 %) compared with never users (8 %, *p* < 0.0001). Calcium channel blocker users had higher BMIs than nonusers (*p* < 0.0001) and were more likely to have used hormone replacement therapy (*p* < 0.0001), both of which are established risk factors for postmenopausal breast cancer. Demographics for users of all classes of antihypertensive drugs compared with nonusers were very similar to calcium channel blocker use alone (Additional file 1: Table S1).

**Table 1:** Study demographic characteristics of never users and ever users of calcium channel blocking drugs at study baseline

	Calcium channel blocker use		
	Never used (*N* = 46,904)	Used (*N* = 3853)	*p* value^a^
Age at baseline	54.5 (48.0–61.0)	60.2 (55.0–66.0)	<0.0001
Age at menarche	12.7 (12.0–13.0)	12.4 (11.5–13.0)	<0.0001
Race			<0.0001
Non-Hispanic White	39,705 (85)	2748 (71)	
Non-Hispanic Black	3655 (8)	797 (21)	
Hispanic	2311 (5)	197 (5)	
Other	1219 (3)	110 (3)	
Missing	14 (<1)	0 (<1)	
Body mass index			<0.0001
<25	18,588 (40)	786 (20)	
25.0–29.9	14,953 (32)	1166 (30)	
30.0–34.9	7839 (17)	944 (25)	
35.0+	5508 (12)	956 (25)	
Missing	16 (<1)	1 (<1)	
Menopause status			<0.0001
Pre menopause	14,254 (30)	461 (12)	
Post menopause	32,332 (69)	3381 (88)	
Missing	318 (1)	11 (<1)	
Ever pregnant			<0.0001
No	5947 (13)	407 (11)	
Yes	40,935 (87)	3445 (89)	
Missing	22 (<1)	1 (<1)	
Ever used hormone replacement therapy			<0.0001
No	26,193 (56)	1532 (40)	
Yes	20,561 (44)	2313 (60)	
Don’t know/missing	150 (<1)	8 (<1)	
Ever diagnosed with cancer^b^			0.0009
No	44,612 (95)	3613 (94)	
Yes	2241 (5)	230 (6)	
Don’t know/missing	51 (<1)	10 (<1)	
Smoking status			<0.0001
Never smoked	26,441 (56)	2045 (53)	
Past smoker	16,604 (35)	1487 (39)	
Current smoker	3845 (8)	318 (8)	
Don’t know/missing	14 (1)	3 (<1)	

Data presented as mean (interquartile range) of *N* (%)

^a^Calculated using chi-square tests and Student’s *t* tests as appropriate

^b^Cancers other than breast cancer (excluding basal-cell carcinoma)

Of the 3844 women in the cohort who reported current use of calcium channel blockers (Table 2), 820 had been using calcium channel blockers for 10 years or more. Among users of calcium channel blockers, women with longer durations of calcium channel blocker use were more likely than short-term users to be postmenopausal or to have ever used hormonal replacement therapy, both of which increase breast cancer risk (Additional file 1: Table S2).

**Table 2:** Use of antihypertensive drugs by breast cancer status

Antihypertensive use	Noncases	Invasive	In situ	Invasiveness unknown	*p* value
	48,792	1372	558	35	
All antihypertensive drugs					
Never used	32,413 (66.4)	883 (64.3)	375 (67.2)	18 (51.4)	0.32
Former user	1204 (2.5)	33 (2.4)	13 (2.3)	1 (2.9)	
Current user (<5 years)	7410 (15.2)	208 (15.1)	92 (16.5)	5 (14.3)	
(5–10 years)	4033 (8.3)	128 (9.3)	40 (7.2)	5 (14.3)	
(10+ years)	3732 (7.6)	120 (8.8)	38 (6.8)	6 (17.1)	
Calcium channel blockers					
Never used	45,089 (92.4)	1270 (92.6)	515 (92.3)	30 (85.7)	0.41
Former user	516 (1.1)	15 (1.1)	6 (1.1)	0 (0)	
Current user (<5 years)	1804 (3.7)	44 (3.2)	21 (3.8)	1 (2.9)	
(5–10 years)	771 (1.6)	25 (1.8)	11 (2.0)	2 (5.7)	
(10+ years)	612 (1.3)	18 (1.3)	5 (0.9)	2 (5.7)	

Data presented as *N* (%)

Distribution of the duration of use for any antihypertensive drug and for use of calcium channel blockers specifically. We calculated distribution of use for women who remained breast cancer free as well as those who developed breast cancer during the study follow-up period

A total of 1965 women reported a breast cancer diagnosis during study follow-up: 1372 cases of invasive breast cancer, and 558 cases that were classified as in situ, and 35 cases in which invasiveness of the tumor was unknown. The mean follow-up time for participants in the cohort was 5.3 years (SD 1.69). Of invasive cancers, 1027 were classified as invasive ductal carcinomas and 130 were invasive lobular carcinomas. Of the 558 in-situ cases, 459 were characterized as ductal carcinoma in situ (DCIS).

Calcium channel blocker use patterns and use of antihypertensive drugs in general did not differ between women who developed breast cancer during follow-up and those who remained free of breast cancer (Table 2). Of women who remained cancer free, 1.3 % had been users of calcium channel blockers for 10 years; this same percentage of women with long-term calcium channel blocker use (1.3 %) was observed among women who developed invasive breast cancer. There were no differences in the distribution of drug usage across cases and noncases for any other subclass of antihypertensive drug (Additional file 1: Table S3).

**Fig. 1:**
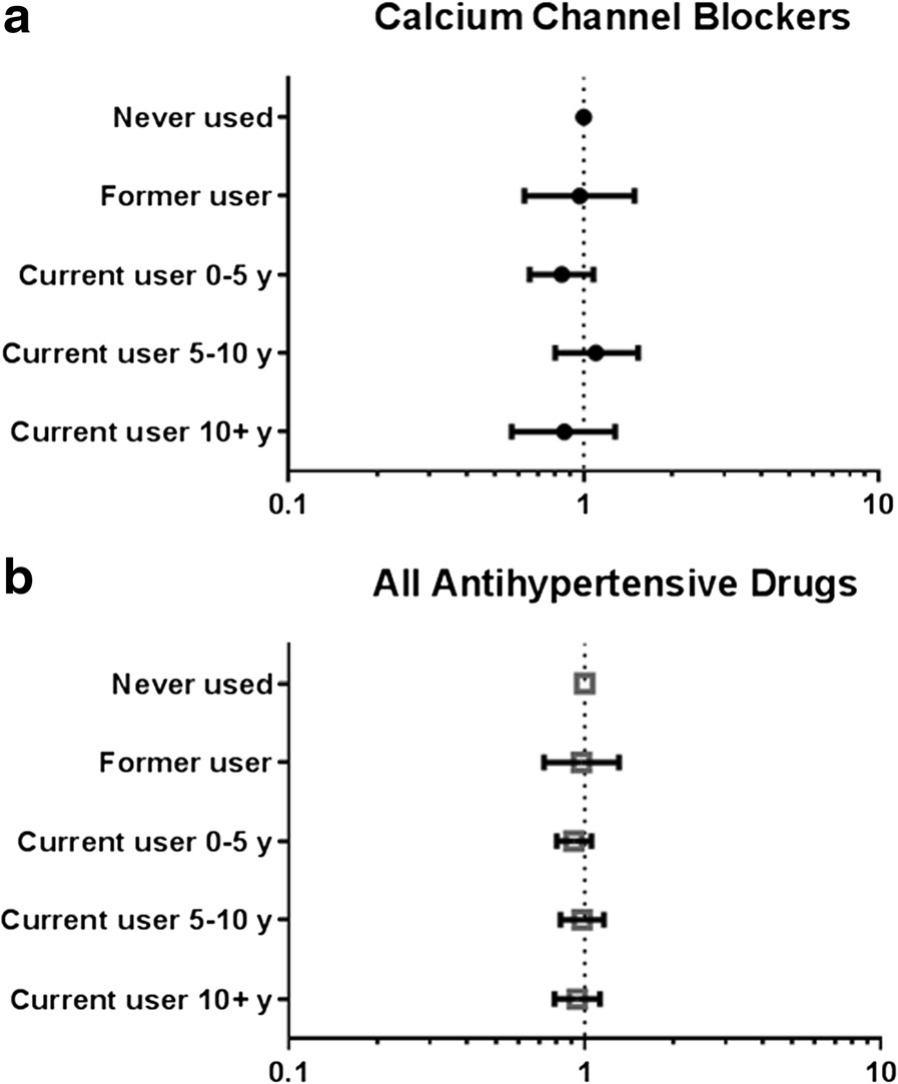
Hazard ratios of incident breast cancer by strata of calcium channel blocker use (**a**) and by strata of use of all antihypertensive drugs (**b**). HRs were calculated using Cox proportional hazards regression with age as the time scale and adjusted for race/ethnicity, categorized BMI, parity, age at menarche, menopause status, statin use, smoking status, hormone therapy use, and reported hours of physical activity per week

Cox proportional hazards regression showed no evidence of increased breast cancer risk with long-term calcium channel blocker use either in unadjusted models (Additional file 1: Table S4) or with adjustment for covariates (Fig. 1). Unadjusted models utilized 50,751 women with 1959 breast cancer events. Owing to missing covariate or follow-up time data, adjusted models resulted in an analytic cohort of 50,269 women, with 1933 breast cancer events. There was no increased risk of breast cancer for any duration of calcium channel blocker use when compared with women who had never used calcium channel blockers (Fig. 1). Being a current user of calcium channel blockers for 10 years or more did not increase risk of breast cancer development during follow-up compared with women who had never used calcium channel blockers (HR 0.88, 95 % CI 0.59–1.33). There was no increased risk observed when long-term use was less strictly categorized as 5 years or more of calcium channel blocker use compared with never users (HR 1.016, 95 % CI 0.78–1.32). Further stratified analysis of dihydropyridine calcium channel blockers and nondihydropyridine calcium channel blockers failed to show increased risk for either class (Additional file 1: Table S5). We also explored antihypertensive use and breast cancer risk more generally, looking at use of any antihypertensive drug (Fig. 1) and various subclasses (Additional file 1: Table S6), but found no evidence of association.

We also explored whether antihypertensive use was associated with postmenopausal breast cancers or with specific tumor subtypes. Analyses restricted to postmenopausal women (*N* = 38,604), women aged over 55, or stratification by ethnicity revealed no increased risk for calcium channel blocker use (Additional file 1: Figure S1). We also conducted analyses stratified by various tumor subtypes. We saw no increased risk for ER+ or ER– tumors only, nor when stratified by invasiveness status at diagnosis (Additional file 1:Supplemental Figure S1). We saw no increased risk for invasive ductal carcinoma (*N* = 1009 cases), for invasive lobular carcinoma (*N* = 130 cases), or for ductal carcinoma in situ (*N* = 459 cases), which were the most common types of incident breast cancers reported during follow-up (Table 3). When the analytical pool was limited to current users of antihypertensive drugs with at least 5 years of use (*N* = 8057) or with at least 10 years of use (*N* = 3879), no increased risk of breast cancer was observed with calcium channel blocker use compared with users of the other classes of antihypertensive drugs (Table 4).

**Table 3:** Hazard ratios for invasive ductal carcinomas, invasive lobular carcinomas, and ductal carcinomas in situ

	Invasive ductal carcinoma (1009 cases)	Invasive lobular carcinoma (130 cases)	Ductal carcinoma in situ (459 cases)
Calcium channel blockers			
Never used	Reference	Reference	Reference
Former user	1.05 (0.59–1.86)	1.68 (0.41–6.85)	0.98 (0.41–2.38)
Current user (<5 years)	0.66 (0.45–0.97)	2.26 (1.12–4.55)	0.78 (0.46–1.35)
(5–10 years)	1.05 (0.66–1.66)	1.13 (0.28–4.61)	1.28 (0.68–2.41)
(10+ years)	0.80 (0.45–1.42)	0.69 (0.10–4.99)	0.80 (0.33–1.94)
All antihypertensives			
Never used	Reference	Reference	Reference
Former user	1.03 (0.69–1.53)	0.30 (0.04–2.16)	1.15 (0.66–2.01)
Current user (<5 years)	0.90 (0.74–1.08)	0.88 (0.51–1.50)	1.06 (0.82–1.39)
(5–10 years)	0.97 (0.77–1.22)	1.21 (0.65–2.24)	0.87 (0.60–1.26)
(10+ years)	0.94 (0.74–1.19)	0.57 (0.24–1.34)	0.88 (0.60–1.28)

Data presented as hazard ratio (95 % confidence interval)

Hazard ratios were calculated using Cox proportional hazards regression with age as the time scale and adjusted for race/ethnicity, categorized body mass index, parity, age at menarche, menopause status, statin use, smoking status, hormone therapy use, and reported hours of physical activity per week

**Table 4:** Hazard ratios of incident breast cancer by strata of calcium channel blocker use in long-term antihypertensive users

Calcium channel blockers	Limited to women currently using an antihypertensive drug for 5 years or more (*N* = 8057)	Limited to women currently using an antihypertensive drug for 10 years or more (*N* = 3879)
Never used	Reference	Reference
Former user	0.87 (0.36–2.11)	0.80 (0.20–3.24)
Current user (<5 years)	1.06 (0.71–1.58)	1.09 (0.60–1.99)
(5–10 years)	1.14 (0.81–1.61)	1.15 (0.58–2.29)
(10+ years)	0.89 (0.58–1.36)	0.89 (0.57–1.40)

Data presented as breast cancer hazard ratio (95 % confidence interval)

Hazard ratios of incident breast cancer by strata of calcium channel blocker use in long-term antihypertensive users when the analytic population is limited to women who have been using an antihypertensive drug for at least 5 years (*N* = 8058) or at least 10 years (*N* = 3879) at baseline. Hazard ratios were calculated using Cox proportional hazards regression with age as the time scale and adjusted for race/ethnicity, categorized body mass index, parity, age at menarche, menopause status, statin use, smoking status, hormone therapy use, and reported hours of physical activity per week

## Discussion

Antihypertensive treatment is on the rise in the USA, with a number of different classes of therapeutic agents available. Calcium channel blockers have become an increasingly common modality of treatment over the past few decades [17, 18]. In light of the increasing prevalence of calcium channel blocker use, recent reports of its potential association with breast cancer could, if substantiated, raise serious questions on how to balance the considerable benefits of this class of agents against potential risks. While a recently conducted case–control study found an association between 10 years or more of calcium channel blocker use and invasive breast cancer [1], our prospective study of 50,757 women with a family history of breast cancer yielded no evidence of increased risk.

There is no strong biological rationale for increased cancer risk resulting from long-term use of calcium channel blockers. It has been hypothesized that use of calcium channel blockers may inhibit intracellular calcium channels involved in signaling and coordinating cell apoptosis [19]. Changes in intracellular calcium concentrations are involved in response pathways to various apoptotic stimuli in many cell types, as well as in the process of DNA fragmentation during apoptosis. Interference with the apoptotic process could potentially allow tumor growth [20–22]. However, in-vivo studies and animal models have shown inconsistent relationships between calcium channel blocking drugs and apoptosis; treatment with the drugs promotes apoptosis in some cancerous and noncancerous cell types and reduces apoptosis in others [23–29]. In fact, calcium channel blocking drugs appear to have antitumorigenic properties in some situations [30–33]. This lack of convincing biological evidence is consistent with the observed lack of association between calcium channel blocker use and breast cancer risk in this study.

The study by Li et al. [1] was a large case–control study of women comprising 880 invasive ductal carcinoma cases, 1027 invasive lobular carcinoma cases, and 856 controls. Their exposure surveys collected detailed information on the use and duration of various classes of antihypertensive drugs. Detailed information on duration of use for subclasses of antihypertensive drugs has not been available in most contemporary studies of breast cancer, so this finding was particularly concerning. However, retrospectively collected exposure information may be subject to recall bias between women with cancer and the population-based controls. The Sister Study’s medication questionnaire method collected very similar information; however, the questionnaires in the Sister Study were administered prospectively, before any diagnosis of breast cancer occurred. Additionally, the observed risk increases in the study be Li et al. were based on a total of 70 women reporting 10 years or more of calcium channel blockers use (*N* = 12 controls, 27 invasive ductal carcinoma, and 31 invasive lobular carcinoma). The small number of long-term users of the calcium channel blocker subclass resulted in wide CI estimates.

Our observed lack of association between calcium channel blocker use and breast cancer risk in this Sister Study is consistent with the results of recently published analyses from the Nurse’s Health Study [12] and the Swedish National Board of Health and Welfare [13]. Both of these studies used prospectively collected data from large cohorts, either from questionnaires or from Swedish Prescription Drug registries which track drug dispensations. Although the Swedish study did not have sufficient numbers of users with 10 years or more of calcium channel blocker use to evaluate risk for those women, neither study found any increased risk of breast cancer development with ever having used calcium channel blockers. We are able to extend these findings to women who are known to be at a higher risk by virtue of having an affected sister, providing additional weight to the argument that calcium channel blockers do not increase breast cancer risk.

Although a small number of women may change medications after enrollment questionnaire assessment, such changes would not be expected to bias risk estimates which are based solely on medication use up to the time of enrollment. This analysis is also limited by a relatively short mean follow-up time of 5.3 years.

While there was no association between calcium channel blocker use and development of breast cancer, we did observe that calcium channel blocker use, and use of antihypertensives in general, is associated with several breast cancer risk factors. Women who used calcium channel blockers in our study were more likely to have a history of smoking, to be overweight, to be postmenopausal, and to have used hormone replacement therapy. These associations might help to explain the inconsistencies observed in past studies of breast cancer risk and antihypertensive drug use. Incomplete adjustment for the differences in risk profiles between calcium channel blocker users and nonusers might produce associations between calcium channel blocker use and breast cancer, particularly in smaller studies.

## Conclusions

Reports of potential excess risk due to antihypertensive drugs are likely to be of particular concern for women with increased breast cancer risk from genetic or other factors. Such concern might lead to less effective treatment for hypertension. Our findings in women with a family history of breast cancer and the findings from other recent prospective studies indicate that calcium channel blocking drugs can be used for long-term hypertension treatment without concern about increases in breast cancer risk.

## Abbreviations

ACE, angiotensin-converting enzyme; BMI, body mass index; CI, confidence interval; DCIS, ductal carcinoma in situ; ER–, estrogen receptor negative; ER+, estrogen receptor positive; HR, hazard ratio; LMP, last menstrual period; SR, sustained release; XR, extended release

## Additional file

Additional file 1: Presents Supplemental Tables S1–S6 and Supplemental Figure S1. (DOCX 286 kb)
