# Supplementary material for: Long-term use of calcium channel blocking drugs and breast cancer risk in a prospective cohort of US and Puerto Rican women
Source: Breast Cancer Res. 2016 Jul 5;18:61. doi: 10.1186/s13058-016-0720-6 (PMC4932734; doi:10.1186/s13058-016-0720-6)
Supplement: Additional file 1: — Presents Supplemental Tables S1–S6 and Supplemental Figure S1. (DOCX 286 kb) [file 13058_2016_720_MOESM1_ESM.docx]

|  | **Antihypertensive Use** | |  |
| --- | --- | --- | --- |
|  |  | |  |
|  | **Never used AHT**  N=33689 | **Used AHT**  N=17068 | P value |
| **Age at Baseline, Mean (IQR)** | 53.3 (47-59) | 59.0 (53-65) | <.0001 |
| **Age at Menarche, Mean (IQR)** | 12.7 (12-13.5) | 12.5 (12-13) |  |
|  |  |  |  |
|  | **N(%)** | **N(%)** |  |
| **Race** |  |  | <.0001 |
| Non-Hispanic White | 28946 (86) | 13507 (79) |  |
| Non-Hispanic Black | 2184 (6) | 2268 (13) |  |
| Hispanic | 1682 (5) | 826 (5) |  |
| Other | 866 (3) | 463 (3) |  |
| Missing | 13(0) | 34(0) |  |
| **BMI** |  |  | <.0001 |
| <25 | 15766 (46) | 3808 (23) |  |
| 25.0-29.9 | 10698 (32) | 5421 (32) |  |
| 30.0-34.9 | 4753 (14) | 4030 (24) |  |
| 35.0+ | 2660 (8) | 3803 (22) |  |
| Missing | 11 (0) | 6 (0) |  |
| **Menopause status** |  |  | <.0001 |
| No | 12208(36) | 2507 (15) |  |
| Yes | 21211 (63) | 14502 (85) |  |
| Missing | 270 (1) | 59 (0) |  |
| **Ever pregnant** |  |  | <.0001 |
| No | 4475 (13) | 1879 (11) |  |
| Yes | 29192 (87) | 15188 (89) |  |
| Missing/Don’t know | 22 (<1) | 1 (<1) |  |
| **Ever used hormone replacement therapy** |  |  | <.0001 |
| No | 20568 (61) | 7157 (42) |  |
| Yes | 13016 (39) | 9858 (58) |  |
| Don't know/missing | 105 (0) | 53 (0) |  |
| **Ever diagnosed with cancer** |  |  | <.0001 |
| No | 32204 (96) | 16021 (94) |  |
| Yes | 1457 (4) | 1014 (6) |  |
| Don't know/Missing | 28(0) | 33 (0) |  |
| **Smoking status** |  |  | <.0001 |
| Never smoked | 18549 (55) | 8789 (51) |  |
| Social smoker | 831 (2) | 318 (2) |  |
| Past smoker | 11522 (34) | 6569 (38) |  |
| Current smoker | 2776 (8) | 1387 (8) |  |
| Don't know/missing | 11 (0) | 5 (0) |  |

Table S1: Demographic characteristics of never users (N=33689) and ever users (N=17068) of antihypertensive drugs at study baseline. P values were calculated using Chi-square tests and student’s t-tests as appropriate.

|  | **Years used Calcium Channel Blockers** | | | | **P value** | |
| --- | --- | --- | --- | --- | --- | --- |
|  | **Never User**  (N=46595) | **0-5 years**  (N=1770) | **5-10 years**  (N=847) | **≥10 years**  (N=756) | **Never vs. Ever used** | **Trend- years of use** |
| **Age at Baseline,**  **Mean (IQR)** | 54.5 (48.0-61.0) | 59.7 (54.0-66.0) | 60.4 (55.0-67.0) | 62.4 (58.0-68.0) | <.0001 |  |
| **Age at Menarche, Mean (IQR)** | 12.7 (12.0-13.0) | 12.5 (12.0-13.0) | 12.4 (12.0-13.0) | 12.4 (11.0-13.0) | <.0001 |  |
|  |  |  |  |  |  |  |
|  | N (%) | N (%) | N (%) | N (%) |  |  |
| **Race** |  |  |  |  | <.0001 | 0.67 |
| Non-Hispanic White | 39710 (85) | 1482 (72) | 680 (71) | 581 (71) |  |  |
| Non-Hispanic Black | 3659 (8) | 419 (20) | 201 (21) | 173 (21) |  |  |
| Hispanic | 2311 (5) | 112 (5) | 47 (5) | 38 (5) |  |  |
| Other | 1219 (3) | 56 (3) | 26 (3) | 28 (3) |  |  |
| Missing | 7 (0) | 1 (0) | 0 (0) | 0 (0) |  |  |
| **BMI** |  |  |  |  | <.0001 | 0.09 |
| <25 | 18590 (39) | 415 (20) | 185 (20) | 184 (23) |  |  |
| 25.0-29.9 | 14958 (32) | 620 (30) | 276 (29) | 265 (32) |  |  |
| 30.0-34.9 | 7840 (17) | 516 (25) | 241 (25) | 186 (23) |  |  |
| 35+ | 5509 (11) | 518(25) | 252 (26) | 185 (23) |  |  |
| Missing | 16 (0) | 1 (0) | 0 (0) | 0 (0) |  |  |
| **Menopause Status** |  |  |  |  | <.0001 | 0.0002 |
| Pre-menopause | 14255 (36) | 291 (14) | 107 (11) | 62 (8) |  |  |
| Post-menopause | 32340 (77) | 1770 (86) | 847 (89) | 756 (92) |  |  |
| Missing | 318 (1) | 9 (0) | 0 (0) | 2 (0) |  |  |
| **Ever Pregnant** |  |  |  |  | <.0001 | 0.32 |
| No | 5947 (13) | 203 (10) | 118 (12) | 86 (10) |  |  |
| Yes | 40944 (87) | 1866 (90) | 836 (88) | 734 (90) |  |  |
| Missing/Don’t know | 22 (0) | 1 (0) | 0 (0) | 0 (0) |  |  |
| **Ever HRT** |  |  |  | 0 (0) | <.0001 | <0.001 |
| No | 26197 (56) | 877 (42) | 377 (40) | 274 (33) |  |  |
| Yes | 20566 (44) | 1186 (57) | 577 (60) | 545 (66) |  |  |
| Missing/Don't know | 150 (0) | 7 (0) | 0 (0) | 1 (0) |  |  |
| **Smoking Status** |  |  |  |  | <.0001 | 0.43 |
| Never smoked | 25360 (54) | 1046 (51) | 502 (53) | 430 (52) |  |  |
| Social smoker | 1086 (2) | 41 (2) | 12 (1) | 10 (1) |  |  |
| Past smoker | 16606 (35) | 808 (39) | 366 (38) | 311 (38) |  |  |
| Current smoker | 3847 (8) | 173 (8) | 74 (8) | 69 (8) |  |  |
| Missing/Don’t know | 14 (0) | 2 (0) | 0 (0) | 0 (0) |  |  |

Table S2: Demographic characteristics across duration of current calcium channel blocker use at study baseline. P values were calculated using Chi-square tests and student’s t-tests as appropriate. Trend p-values were calculated using univariate multinomial logistic regression.

| Antihypertensive Use | Non-cases  N (%) | Invasive  N (%) | In situ  N (%) | Invasiveness Unknown N(%) | P trend |
| --- | --- | --- | --- | --- | --- |
| **Beta-blockers** |  |  |  |  |  |
| Never used | 42351 (86.0) | 1175 (85.6) | 478 (85.7) | 26 (74.3) |  |
| Former user | 1017 (2.1) | 30 (2.2) | 10 (1.8) | 1 (2.9) |  |
| Current user (<5 years) | 2832 (5.8) | 89 (6.5) | 45 (8.1) | 3 (8.6) |  |
| (5-10 years) | 1348 (2.8) | 37 (2.7) | 13 (2.3) | 1 (2.9) |  |
| (10+ years) | 1244 (2.5) | 41 (3.0) | 12 (2.1) | 4 (11.4) | 0.27 |
| **ACE inhibitors** |  |  |  |  |  |
| Never used | 43691 (89.5) | 1226 (89.4) | 511 (91.6) | 29 (82.9) |  |
| Former user | 651 (1.3) | 12 (0.9) | 5 (0.9) | 0 (0) |  |
| Current user (<5 years) | 2621 (5.4) | 73 (5.3) | 30 (5.4) | 2 (5.7) |  |
| (5-10 years) | 1085 (2.2) | 39 (2.8) | 7 (1.3) | 3 (8.6) |  |
| (10+ years) | 754 (1.6) | 22 (1.6) | 5 (0.9) | 1 (2.8) | 0.22 |
| **Angiotensin receptor blockers** |  |  |  |  |  |
| Never used | 44706 (91.6) | 1247 (90.9) | 510 (91.4) | 33 (94.3) |  |
| Former user | 317 (0.7) | 11 (0.8) | 3 (0.5) | 0 (0) |  |
| Current user (<5 years) | 2600 (5.3) | 74 (5.4) | 32 (5.7) | 1 (2.9) |  |
| (5-10 years) | 813 (1.7) | 31 (2.3) | 8 (1.4) | 1 (2.9) |  |
| (10+ years) | 356 (0.7) | 9 (0.7) | 5 (0.9) | 0 (0) | 0.93 |
| **Diuretics** |  |  |  |  |  |
| Never used | 40479 (83.0) | 1110 (80.9) | 451 (80.8) | 22 (62.8) |  |
| Former user | 761 (1.6) | 24 (1.7) | 8 (1.4) | 0 (0) |  |
| Current user (<5 years) | 3964 (8.1) | 118 (8.6) | 53 (9.5) | 5 (14.3) |  |
| (5-10 years) | 1892 (3.9) | 61 (4.4) | 19 (3.4) | 3 (8.6) |  |
| (10+ years) | 1696 (3.5) | 59 (4.3) | 27 (4.8) | 5 (14.3) | 0.12 |

Table S3: Distribution of duration of use for additional subclasses of antihypertensive drugs. We calculated distribution of use for women who remained breast cancer free as well as those who developed breast cancer during the study follow-up period.

|  | **Breast Cancer** | **Invasive ductal carcinoma only** |
| --- | --- | --- |
|  | Hazard Ratio (95% CI) | Hazard Ratio (95% CI) |
| **All Antihypertensives** |  |  |
| Never used | ref | ref |
| Former user | 0.97 (0.72-1.29) | 1.02 (0.69-1.50) |
| Current user (<5 years) | 0.94 (0.83-1.07) | 0.90 (0.76-1.08) |
| (5-10 years) | 0.99 (0.84-1.16) | 0.97 (0.77-1.21) |
| (10+ years) | 0.96 (0.81-1.13) | 0.96 (0.76-1.21) |
| **Calcium channel blockers** |  |  |
| Never used | ref | ref |
| Former user | 0.97 (0.63-1.49) | 1.02 (0.58-1.81) |
| Current user (<5 years) | 0.84 (0.66-1.07) | 0.63 (0.43-0.93) |
| (5-10 years) | 1.11 (0.80-1.53) | 1.02 (0.65-1.61) |
| (10+ years) | 0.90 (0.60-1.33) | 0.78 (0.44-1.38) |

Table S4: Unadjusted hazard ratios of incident breast cancer by strata of antihypertensive use and calcium channel blocker use only. Hazard ratios were calculated using Cox proportional hazards regression with age as the time scale.

|  | **Any calcium channel blocker** | **Non-dihydropine CCB** | **Dihydropine CCB** |
| --- | --- | --- | --- |
|  | Hazard Ratio of breast cancer (95% CI) | Hazard Ratio of breast cancer (95% CI) | Hazard Ratio of breast cancer (95% CI) |
| **Calcium channel blocker use** |  |  |  |
| Never used | ref | ref | ref |
| Former user | 0.97 (0.63-1.49) | 0.87 (0.47-1.62) | 1.06 (0.62-1.79) |
| Current user (<5 years) | 0.84 (0.66-1.08) | 1.08 (0.73-1.59) | 0.78 (0.57-1.06) |
| (5-10 years) | 1.10 (0.80-1.53) | 0.99 (0.57-1.71) | 1.17 (0.79-1.74) |
| (10+ years) | 0.86 (0.57-1.28) | 1.08 (0.66-1.77) | 0.61 (0.30-1.23) |

Table S5: Hazard ratios for breast cancer across strata of calcium channel blocker use, stratified by non-dihydropine calcium channel blockers and dihydropine calcium channel blockers. Hazard ratios were calculated using Cox proportional hazards regression with age as the time scale.

| Antihypertensive Use | Hazard Ratio of breast cancer  (95% CI) | Hazard Ratio of invasive breast cancer (95% CI) |
| --- | --- | --- |
| **Beta-blockers** |  |  |
| Never used | Ref. | Ref. |
| Former user | 0.96 (0.72-1.34) | 1.02 (0.71-1.47) |
| Current user (<5 years) | 1.07 (0.89-1.28) | 0.97 (0.77-1.21) |
| (5-10 years) | 0.87 (0.65-1.15) | 0.87 (0.63-1.21) |
| (10+ years) | 0.98 (0.74-1.284) | 0.97 (0.71-1.34) |
| **ACE inhibitors** |  |  |
| Never used | Ref. | Ref. |
| Former user | 0.56 (0.34-0.92) | 0.55 (0.31-1.00) |
| Current user (<5 years) | 0.92 (0.75-1.13) | 0.92 (0.72-1.17) |
| (5-10 years) | 1.02 (0.76-1.36) | 1.14 (0.82-1.57) |
| (10+ years) | 0.83 (0.57-1.21) | 0.91 (0.60-1.40) |
| **Angiotensin receptor blockers** |  |  |
| Never used | Ref. | Ref. |
| Former user | 1.12 (0.66-1.89) | 1.24 (0.69-2.25) |
| Current user (<5 years) | 0.93 (0.76-1.14) | 0.90 (0.71-1.15) |
| (5-10 years) | 1.19 (0.87-1.64) | 1.29 (0.90-1.85) |
| (10+ years) | 0.93 (0.55-1.58) | 0.83 (0.43-1.60) |
| **Diuretics** |  |  |
| Never used | Ref. | Ref. |
| Former user | 1.02 (0.71-1.44) | 1.08 (0.72-1.62) |
| Current user (<5 years) | 1.03 (0.87-1.21) | 0.96 (0.79-1.17) |
| (5-10 years) | 1.03 (0.83-1.30) | 1.07 (0.82-1.39) |
| (10+ years) | 1.18 (0.95-1.47) | 1.09 (0.83-1.42) |

Table S6: Hazard ratios for other subclasses of antihypertensive drugs calculated using Cox proportional hazards regression with age as the time scale. Models were adjusted for baseline reported race/ethnicity, categorized BMI, parity, age at menarche, menopause status history of breast conditions, statin use, smoking status, hormone therapy use, and reported hours of physical activity per week.

Menopause status Tumor invasiveness Tumor estrogen receptor status

Figure S1: Hazard ratios of incident breast cancer by strata of calcium channel blocker use stratified by A.) Menopause status at study baseline: 35595women post-menopause and 14675 women pre-menopause . B) Outcome tumor characteristics: 1372 invasive tumors and 593 in situ tumors; C) Outcome tumor estrogen receptor status: 1402 estrogen receptor positive tumors and 290 estrogen receptor negative tumors
